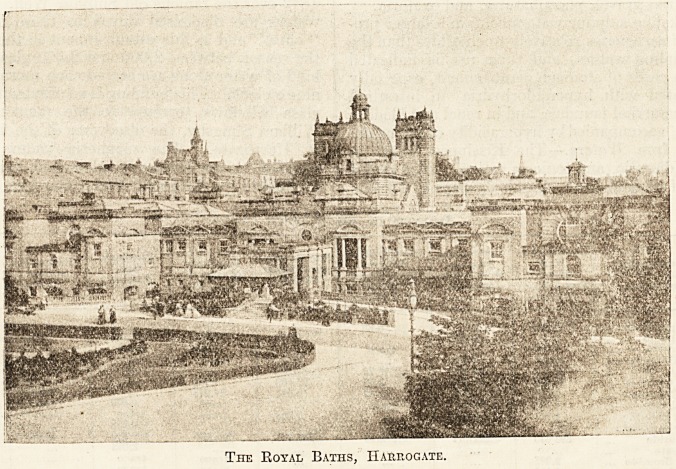# Home and Continental Spas
*Previous articles in this series appeared in The Hospital Jan. 28, Feb. 25, March 25, and April 22.


**Published:** 1911-05-20

**Authors:** 


### HOME AND CONTINENTAL SPAS.*

### V.—HARROGATE.

No Spa in this country possesses a more varied selection of mineral springs than Harrogate, while the Corporation of the town has done everything to supplement the natural advantages and so to render them of greater efficacy in the alleviation of disease. The high esteem in which the waters are regarded is shown by the fact that the medical advisers to the Royal Family have on several occasions recommended a visit of some member to this particular Spa. Only last July Princess Victoria was here for nearly a month, taking the "cure" with the most satisfactory results.

Harrogate is pleasantly situated in the West Riding district of Yorkshire, on a plateau some 400 to 500 feet above sea-level, and surrounded by extensive moorland, which is doubtless responsible for the peculiar "freshness" of the air, a fact at once evident to the visitor. The town is well planned, with fine, broad streets and an abundance of open places, whilst the many handsome public buildings give the Spa a most imposing appearance. A unique feature is the Stray, a broad expanse of common land, some 200 acres in extent, which runs through the centre of the town. This is intersected by paths in all directions and, being well supplied with seats, is a favourite resort in the spring and summer months. The Spa, which is 200 miles from London, is reached in about four hours from King's Cross station by the Great Northern and North Eastern Railways' express service, and as most of the trains are equipped with corridor carriages and restaurant cars, the journey is made in the greatest possible comfort.

THE ROYAL BATHS, HARROGATE.

The population has steadily increased during the last twenty years, and there are now over 33,000 inhabitants. In the height of the season, which extends from May to October, it is estimated that the population reaches 60,000 persons. The occurrence of disease among the residents of the Spa is below the average, while epidemics are exceptionally rare. This is probably due to the fact that Harrogate is one of the best-drained towns in this country, the sewerage system being on the irrigation principle combined with bacterial treatment. A thoroughly efficient drainage scheme, by which the sewage is carried some miles beyond the town, has recently been completed by the Corporation, at a cost of £165,000. The domestic water-supply is derived from the extensive watershed at Haverah Park, Scargill, and the adjacent moors, far removed from all possible source of contamination.

The climate is bracing and dry, a condition attributed to the amount of sunshine and to the rapid evaporation and thorough drainage. The average hours of sunshine taken over a period of ten years is 1640.6, which shows Harrogate to be one of the sunniest places in the Kingdom. The average annual rainfall for the past thirty-eight years is 29.54 inches, and the average number of rainy days 195, of which at least 10 per cent. may be taken as rainy days when less than 0.02 inch has fallen during the 24 hours. In consequence of the absence of manufactories in the neighbourhood, the Spa is singularly free from smoke pollution and fog.

The mineral waters of Harrogate are exceptional both in number and variety, there being no fewer than eighty known springs differing in quality and

*Previous articles in this series appeared in THE HOSPITAL Jan. 28, Feb. 25, March 25, and April 22.

strength. Of these, sixteen are used for internal administration, the remainder being employed for bathing purposes. These waters may be divided into two distinct groups, namely, sulphur waters and iron waters, and of each group there are several varieties, as will be seen from the analyses.

*The Sulphur Waters.*—The medicinal action of these waters depends greatly upon the particular water prescribed, its quantity, time when taken, and temperature. They are generally laxative, aperient or purgative, diuretic or alterative. The stronger sulphur waters are of assistance in the treatment of functional disorders of the liver, gout, chronic rheumatoid arthritis, muscular rheumatism, gall-stones, and catarrhal affections of the stomach and intestines. They are also used in obesity and glycosuria, anæmia of toxic origin, plumbism and mercurial poisoning, and chronic skin diseases. The milder sulphur waters are employed in similar diseases where the patient is delicate or debilitated. The magnesia water is diuretic in action and is employed to supplement the action of the stronger waters and to eliminate the products of faulty metabolism. It is also beneficial in glycosuria, gouty albuminuria, gravel, phosphaturia, and oxaluria.

The alkaline sulphur waters contain a larger proportion of carbonates relatively to chlorides than the other sulphur waters, and their use is indicated mainly in cases of stomach derangement, especially if associated with hyperchlorhydria, in intestinal catarrh, catarrhal jaundice, and in renal and bladder affections, accompanied by hyperacidity of the urine.

*Saline Iron Waters.*—The Kissingen water is aperient, diuretic, and tonic in action. It is useful in cases of sluggish liver, chronic dyspepsia, nervous disorders, such as hysteria and neurasthenia, in debility following prolonged illness, and in anæmia, when supplemented by a strong chalybeate water. The chloride of iron water is one of the strongest known natural iron waters and contains, in addition to the carbonate and chloride of iron, the powerful drug, barium chloride. It is a valuable tonic and is beneficial in chlorosis and all forms of anæmia, nervous debility, tuberculous affections, especially of glands, and in all chronic cachectic conditions.

*Pure Chalybeate Waters.*—These are mild iron tonics, used principally in cases of anæmia. They are quite suitable for administration to young children and may be given not only for their tonic effect, but, when indicated, in larger doses for their diuretic action.

### The Pump Rooms.

There are three establishments where the mineral waters may be obtained, the principal of which is the Royal Baths pump room in the central hall of the Royal Baths, but the most popular is the Royal Pump Room, a conspicuous octagonal structure in the lower part of the town. This was erected in 1842 over the famous old Sulphur Well. Here the waters are dispensed direct to those taking the "cure," and at this establishment in the height of the season between 2,000 and 2,500 glasses of one kind of water alone are served each morning before nine o'clock. This building is adorned with stained-glass windows, erected to the memory of Sir William Slingsby, the discoverer of the first spring in Harrogate. The magnesia waters may be obtained at the handsome glass and iron building situated in the delightful Valley Gardens.

### Analysis of the Sulphur Group.

| Saline Constituents in Grains per Gallon. | Old Sulphur Well Royal Pump Room (Thorpe). | Strong Sulphur Montpellier (Attfield). | New or Mild Sulphur Royal Pump Room (W.A.Miller). | Mild Sulphur Montpellier (Attfield). | Magnesia (Muspratt). | Starbeck Spa (Fairley). | Beckwith Spring. | No. 36 Sulphur Well. |
|---|---|---|---|---|---|---|---|---|
| Sodium Sulphydrate ... | 5.215 | ... | 6.89 | ... | ... | ... | ... | ... |
| Sodium Sulphide ... | ... | 14.500 | ... | 8.777 | 10.707 | 1.515 | 1.929 | 1.1 |
| Barium Chloride ... | 6.566 | ... | trace | ... | 1.222 | trace | ... | |
| Strontium Chloride ... | trace | 2.816 | ... | 6.19 | trace | ... | ... | ... |
| Calcium Chloride ... | 43.635 | 79.936 | 16.70 | 31.296 | ... | ... | ... | ... |
| Magnesium Chloride ... | 48.281 | 57.939 | 2.39 | 27.589 | 1.792 | ... | ... | ... |
| Potassium Chloride ... | 9.592 | 4.811 | 11.34 | 5.691 | 27.913 | ... | 0.610 | 26.4 |
| Lithium Chloride ... | 0.753 | trace | trace | ... | trace | 0.070 | trace | 0.5 |
| Ammonium Chloride ... | 1,031 | 0·996 | ... | 0.653 | trace | trace | ... | 0.4 |
| Ammonium Carbonate .. | ... | ... | ... | ... | ... | 0.225 | ... | ... |
| Sodium Chloride ... | 893·670 | 827,371 | 582.95 | 338.800 | 215.896 | 109.890 | 3.374 | 241.6 |
| Sodium Silicate ... | ... | ... | ... | ... | ... | 2.073 | ... | ... |
| Magnesium Bromide ... | 2.283 | ... | ... | ... | trace | trace | ... | ... |
| Magnesium Iodide ... | 0.113 | ... | ... | ... | trace | trace | ... | ... |
| Calcium Carbonate ... | 29.768 | 8.750 | ... | 16.711 | 18.476 | 7.825 | 5.947 | 11.1 |
| Magnesium Carbonate ... | 5.953 | ... | ... | ... | 12.799 | 4.119 | 4.222 | 7.2 |
| Ferrous Carbonate ... | ... | ... | ... | ... | ... | 0.072 | ... | ... |
| Potassium Carbonate ... | ... | ... | ... | ... | ... | 1.745 | | 14.529 |
| Sodium Carbonate ... | ... | ... | ... | ... | ... | 17.104 | 16.[illegible]47 | ... |
| Sodium Iodide ... | ... | ... | ... | ... | ... | 0.001 | ... | ... |
| Barium Sulphate ... | ... | 0.418 | ... | ... | ... | ... | ... | ... |
| Barium Carbonate ... | ... | ... | ... | ... | ... | 2.275 | ... | 5.3 |
| Strontium Sulphate ... | ... | 0.529 | ... | 0.913 | ... | ... | ... | ... |
| Strontium Carbonate ... | ... | ... | ... | ... | ... | 0.141 | | ... |
| Calcium Sulphate ... | ... | ... | ... | ... | ... | 1.88 | 0.530 | ... |
| Sodium Nitrate ... | ... | 0.900 | ... | 0.370 | ... | | ... | |
| Silica ... ... ... | 0.701 | 3.570 | 2.40 | 3.836 | 1.608 | 3.27 | 0.570 | 10.7 |
| | 1047.561 | 1002.586 | 654.87 | 485.253 | 280.413 | 151.59 | 33.339 | 300.4 |
| Gases in Cubic Inches. | | | | | | | | |
| Sulphuretted Hydrogen | 10.46 | ... | 4.18 | ... | ... | 1.78 | ... | 5.6 |
| Carbon Dioxide ... | 40.10 | 60.00 | 13.22 | 54.00 | 11.50 | 2.71 | ... | 30.5 |
| Carburetted Hydrogen | ... | 2.30 | ... | 0.80 | ... | ... | ... | ... |
| Nitrogen ... ... ... | ... | 3.70 | 2.01 | 3.20 | ... | 6.34 | ... | ... |
| | 50.56 | 66.00 | 19.41 | 58.00 | 11.50 | 10.83 | ... | 36.1 |

### The Bathing Establishments.

The Royal and the Victoria Baths belong to the Corporation and are replete with every appliance known to modern hydrotherapy. The enterprising manager, Mr. H. J. Buckland, who has been so instrumental to the success of the Spa, makes periodic visits to the Continental health-resorts in order that Harrogate may possess the very last thing in apparatus and treatment.

The Royal Baths, which is the principal bathing establishment, is a palatial building, well equipped, erected at a cost of £120,000 and opened by his Royal Highness the late Duke of Cambridge on July 23, 1897.

Although the Victoria Baths is the original bathing establishment, it has been thoroughly adapted to modern requirements, and the treatment administered here is in every respect efficient and up to date.

There are also the Starbeck Baths, a mile or two without the town, and easily accessible by train. These were formerly private, but are now the property of the Corporation. They contain, in addition to the ordinary immersion-baths, a swimming-bath of mild sulphur water. No fewer than fifty modes of treatment are to be obtained at one or other of the bathing establishments, among which may be mentioned sulphur baths, massage baths, massage douche, hot air, vapour and electric baths, including Dowsing, Greville, combined light and heat, Berthe, Berthollet, *x*-rays, D'Arsonval high-frequency, electric immersion.

Special attention is devoted to Plombières treatment of muco-membranous colitis, for which certain of the mineral waters have proved to be most efficacious. Owing to the increasing number of patients, considerable extensions have recently been effected in this department.

The treatment includes two procedures—an internal douche for washing out the bowel with the prescribed mineral water, and an immersion bath, with an external "Tivoli" or "Submassive" douche to the abdomen, given under water in the form of a spray at a raised temperature.

Special mention must also be made of the new wing of the Royal Baths which is set aside for treatments of the throat, nose, eye and ear. The appliances here are most perfect and absolutely unique as far as this country is concerned.

### Accommodation.

It would not be easy to find a town in the United Kingdom possessing a greater number of palatial hotels; indeed, it is somewhat difficult to realise how these huge establishments obtain support sufficient to their maintenance in so short a season as from May to October. There are many others more modest in character, besides an abundance of hydros, boarding houses, and apartments, so that the visitor is assured of comfortable accommodation whatever his means, and no one need hesitate to visit Harrogate in consequence of the smallness of his bank balance. As the baths occupy a central position in the town they are easily accessible from all parts, and where walking is inadvisable or impossible, flys, taxicabs and bath chairs may be hired in the principal thoroughfares.

Harrogate is renowned for the amenities of its social life. Music is provided in the season in all the public gardens, and on wet days in the Winter

#### Analysis of the Saline Chalybeate and Iron Group.

| Saline Constituents in Grains per Gallon. | Kissingen Spa (Attfield). | Chloride of Iron Spa (Thorpe). | Alexandra Chalybeate (Davis). | Carbonate of Iron Spa (Muspratt). | Pure Chalybeate Royal Pump (Davis). | Tew't Well (Hofmann). | John Well or Old Spa. | Alum Well (Davis). |
|---|---|---|---|---|---|---|---|---|
| Ferrous Chloride ... | ... | 13.213 | ... | ... | ... | ... | ... | ... |
| Ferrous Carbonate ... | 9.590 | 11.050 | 5.800 | 6.042 | 1.364 | 1.358 | 1 271 | |
| Ferrous Sulphate ... | ... | ... | ... | ... | ... | ... | ... | 69.33 |
| Ferric Sulphate ... ... | ... | ... | ... | ... | ... | ... | ... | 78.73 |
| Aluminium Sulphate ... | ... | ... | ... | ... | ... | ... | ... | 89.47 |
| Calcium Sulphate ... | ... | ... | 9.097 | 7.625 | 0.740 | 0.697 | 0.307 | 56·91 |
| Magnesium Sulphate ... | ... | ... | ... | ... | ... | ... | ... | 67.33 |
| Potassium Sulphate ... | ... | ... | ... | ... | ... | ... | ... | 3.14 |
| Ammonium Sulphate ... | ... | ... | ... | ... | ... | ... | ... | 2.19 |
| Barium Sulphate ... | 0.509 | .222 | ... | ... | ... | ... | ... | ... |
| Potassium Chloride ... | 21.425 | 3·205 | 1.130 | 0.150 | ... | 1.323 | ... | .. |
| Sodium Chloride ... | 674.598 | 277·561 | 176·370 | 11.650 | 1.625 | 0.280 | 1.543 | 33.96 |
| Ammonium Chloride ... | 0.439 | ·406 | trace | ... | trace | trace | trace | ... |
| Barium ... ... ... | ... | 5·204 | ... | ... | ... | ... | ... | ... |
| Strontium Chloride ... | 0 887 | .624 | ... | ... | ... | ... | ... | ... |
| Calcium Chloride ... | 87.337 | 94.015 | ... | 2.311 | . | ... | ... | ... |
| Manganese Chloride ... | ... | .971 | trace | ... | trace | trace | ... | ... |
| Magnesium Chloride ... | 60.391 | 57.315 | 4·736 | 13.148 | ... | ... | ... | ... |
| Lithium Iodides ... | ... | ... | ... | ... | ... | ... | ... | ... |
| Bromides, Fluordides ... | traces | traces | traces | ... | trace | trace | ... | ... |
| Barium Carbonate ... | 2.136 | ... | ... | ... | ... | .. | ... | ... |
| Calcium Carbonate ... | 8.858 | ... | 13·762 | 0.341 | 1.532 | 1.435 | 2.261 | ... |
| Magnesium Carbonate ... | ... | ... | 5·785 | ... | 1.952 | 2.667 | 3.039 | ... |
| Potassium Carbonate ... | ... | ... | ... | ... | 0.262 | 1.0[illegible]7 | 0.991 | ... |
| Sodium Carbonate ... | ... | ... | ... | | 1.103 | ... | 1.338 | ... |
| Silica ... ... ... | 3.[illegible]70 | 1.414 | 5·675 | 0.204 | 0.502 | 1.041 | trace | 3.27 |
| Organic Matter ... ... | ... | ... | 1·450 | ... | 0.[illegible]70 | 0.063 | trace | |
| | 874.740 | 465.200 | 218·804 | 41.471 | 9.839 | 10.521 | 1[illegible]7 3 | 394.41 |
| **Gases in Cubic Inches.** | | | | | | | | |
| Carbon Dioxide ... ... | 21.3 | 53.55 | 17·04 | ... | 13.74 | 11.85 | 14.95 | ... |
| Carburetted Hydrogen... | ... | ... | ... | ... | ... | .. | .15 | ... |
| Oxygen ... ... ... | 1.5 | ... | ·31 | ... | .[illegible]2 | .40 | .67 | ... |
| Nitrogen ... ... ... | 5.2 | ... | 8·98 | ... | 8.00 | 5.33 | 6.35 | ... |
| | 28.0 | ... | 26·33 | ... | 22.56 | 17.78 | 22.12 | ... |

Gardens, a magnificent building adjoining the Royal Baths. The Valley Gardens are, however, the most fashionable rendezvous between the hours of 3.30 and 5 on a summer afternoon. A magnificent palace of entertainment, the Kursaal is situated almost opposite the Royal Baths and contains one of the most beautiful concert halls in the world. A concert is given here every day by the Corporation orchestra, which numbers fifty performers, under the conductorship of Mr. Julian Clifford.

The opportunities for outdoor recreations are numerous and diverse. There are two excellent golf links, pleasantly situated and easily accessible by road or rail. Cricket, lawn tennis, croquet and bowls have their grounds, to which visitors are readily admitted. Excellent boating is to be obtained on the River Nidd and angling in the Crimple and Ure. There are three packs of fox-hounds within easy distance—the Bramham Moor, York and Ainsty, and the Bedale Hounds. As a centre for motoring, driving, and cycling the Spa is exceptionally well situated.

## Figures and Tables

**Figure f1:**